# The Unseen Struggle: Evaluating Compliance With Dysphagia Assessment Guidelines in Hospitalised Parkinson’s Patients

**DOI:** 10.7759/cureus.117736

**Published:** 2026-10-10

**Authors:** Aparna Ravikumar, Nisarg Shah, Riya Raphael, Lucy Meadows

**Affiliations:** 1 Internal Medicine, York and Scarborough Teaching Hospitals NHS Foundation Trust, Scarborough, GBR; 2 Geriatrics, York and Scarborough Teaching Hospitals NHS Foundation Trust, Scarborough, GBR

**Keywords:** dysphagia', multi-disciplinary teams, parkinsons disease, recurrent aspiration pneumonia, swallow screening

## Abstract

Background

What is effortless for most - a simple act of swallowing - can pose a significant and often under-recognised risk for patients with Parkinson’s disease (PD). Dysphagia is frequently overshadowed by the motor and non-motor complexity of PD, yet it contributes substantially to aspiration pneumonia, malnutrition and disrupted medication delivery, particularly during acute hospitalisation. Aspiration pneumonia is recognised as the leading cause of mortality in Parkinson’s disease, underscoring the clinical importance of early identification of swallowing impairment. The National Confidential Enquiry into Patient Outcome and Death (NCEPOD) recommends swallow screening for all acutely unwell patients with Parkinson’s disease and provides National Recommendations aimed at improving the quality and safety of dysphagia care during hospital admission. This audit aimed to evaluate local adherence to NCEPOD recommendations for dysphagia screening and management in hospitalised patients with Parkinson’s disease.

Methods

A retrospective quality-improvement audit was conducted at Scarborough General Hospital, York and Scarborough Teaching Hospitals NHS Foundation Trust. Forty-five acute admissions of patients with a confirmed diagnosis of Parkinson’s disease between May 2023 and December 2024 were reviewed. Electronic patient records, prescribing records, Speech and Language Therapy documentation, nursing assessments and discharge summaries were assessed against NCEPOD recommendations. Audit measures included admission swallow screening, referral to Speech and Language Therapy and Parkinson’s specialist services, timely administration of dopaminergic medication, nutritional screening, multidisciplinary involvement and documentation of swallowing-related information at discharge.

Results

Swallow screening at hospital admission was completed in only 18% (8/45) of cases. Among those screened, swallowing impairment was identified in 88% (7/8), a finding that, while based on a small screened subgroup, highlights the potential for clinically important dysphagia to remain unrecognised when systematic assessment is not routinely performed. During admission, 24% (11/45) developed new or worsening dysphagia; however, only 36% (4/11) were referred to Speech and Language Therapy. Timely dopaminergic medication administration was maintained in 91% (41/45) of admissions, although documentation of alternative medication routes in patients with dysphagia was inconsistent. Nutritional screening was completed in 51% (23/45) of cases, while swallowing-related advice was included in only 7% (3/45) of discharge summaries, highlighting gaps in multidisciplinary communication and continuity of care.

Conclusion

This audit identifies meaningful gaps in the recognition and management of dysphagia among hospitalised patients with Parkinson’s disease. Although swallowing impairment was common when assessed, inconsistent screening and variable escalation limited timely specialist involvement and continuity of care. Increasing awareness of bedside swallow assessments, such as sip tests, alongside a structured swallow checklist at admission, may improve early and consistent identification of swallowing impairment. Standardised multidisciplinary referral pathways and improved documentation, particularly at discharge, may further strengthen continuity of care, reduce aspiration-related risk and support safer, more coordinated management across acute and community settings.

## Introduction

Dysphagia is one of the most distressing yet frequently overlooked non-motor manifestations of Parkinson’s disease (PD) [1]. Existing literature suggests that more than 80% of individuals with PD develop swallowing difficulties during the course of their illness [2-4]. What may initially present as mild dysphagia can progress insidiously, resulting in aspiration pneumonia - which is the leading cause of mortality in Parkinson’s disease, as well as malnutrition and prolonged hospitalisation [1,5].

Beyond nutritional compromise, in acutely unwell patients with Parkinson’s disease, dysphagia can impair the timely delivery of dopaminergic medications, exacerbating motor fluctuations, increasing symptom burden, and contributing to functional decline [6]. The NICE Quality Standard for Parkinson’s disease (QS164) emphasises that missed or delayed doses of levodopa are associated with worsening symptoms, increased risk of serious complications, prolonged hospital stay, and reduced quality of life [7].

The National Confidential Enquiry into Patient Outcome and Death (NCEPOD) report, Hard to Swallow?, outlines several key recommendations to improve the safety and quality of care for patients with Parkinson’s disease [8]. These include performing a swallow screen at the point of admission, initiating prompt referral to Speech and Language Therapy (SLT) when swallowing impairment is identified, and prompt involvement of Parkinson’s disease specialist teams when there are concerns regarding disease progression [8]. Additional recommendations emphasise the importance of assessing nutritional status, ensuring the timely administration of dopaminergic medications throughout admission, and documenting swallowing status clearly in discharge summaries to support continuity of care and reduce preventable complications [8].

This audit aimed to evaluate local adherence to these national recommendations within a district general hospital in the United Kingdom. By identifying both gaps in practice and areas of strength, the project sought to support safer, more consistent, and compassionate inpatient care for individuals with Parkinson’s disease during episodes of acute illness, while identifying opportunities for targeted improvement across the dysphagia care pathway.

## Materials and methods

Study design

A retrospective quality-improvement audit was conducted at Scarborough General Hospital, York and Scarborough Teaching Hospitals NHS Foundation Trust, United Kingdom, over a 20-month period from May 2023 to December 2024. Patients with a confirmed diagnosis of Parkinson’s disease who were admitted acutely during this time were included, resulting in 45 acute admissions representing 45 individual patients identified through electronic patient records. Elective admissions and patients without a documented diagnosis of Parkinson’s disease were excluded. The project was registered with the Trust Clinical Audit Department (QIP038). Ethical approval was not required, as all data were anonymised and collected for service-evaluation purposes.

Data collection and audit parameters

Data were extracted from electronic patient records, electronic prescribing systems (EPMA), Speech and Language Therapy documentation, nursing assessments, and discharge summaries. Audit measures were aligned with the recommendations outlined in the National Confidential Enquiry into Patient Outcome and Death (NCEPOD) report, Hard to Swallow? A Review of the Quality of Dysphagia Care Provided to Patients With Parkinson’s Disease [8], which outlines key recommendations to improve the quality and safety of care for people with Parkinson’s disease when they are acutely unwell. These standards emphasise early recognition of dysphagia and robust multidisciplinary management.

Accordingly, the audit assessed whether swallow status was documented at the point of referral, whether the Parkinson’s disease specialist team was notified when deterioration or disease progression was suspected, and whether all patients received a swallow screen on admission, including evaluation of their ability to swallow food, fluids, and medication, saliva control, and any history of pneumonia. The parameters also examined the timeliness of referral to Speech and Language Therapy (SLT), the safe and timely administration of dopaminergic medications with alternative formulations considered when swallowing was compromised, and the completion of a documented medication review at discharge.

The audit also assessed whether nutritional screening using the Malnutrition Universal Screening Tool (MUST), a five-step screening tool used to identify adults who are malnourished or at risk of malnutrition, had been completed during admission and whether patients identified as being at nutritional risk were subsequently referred to dietetic services [9]. In addition, the audit evaluated whether multidisciplinary involvement, including SLT, pharmacy, dietetics, and nutrition teams, occurred when swallowing difficulties were present and whether discharge summaries provided clear information regarding swallow status and medication considerations.

Together, these measures reflect national recommendations aimed at strengthening continuity of care, reducing avoidable harm, and improving the quality and safety of dysphagia-related care for hospitalised patients with Parkinson’s disease. The audit parameters are summarised in Table 1.

**Table 1:** Audit Parameters Original table created by the authors to summarise the audit standards, which were based on recommendations and guidance from the National Confidential Enquiry into Patient Outcome and Death (NCEPOD) for improving the quality of care for people with Parkinson’s disease and dysphagia [8].

Parameters Analyzed
1. Was the patient’s swallow status documented by the GP or community team at the point of referral?
2. Was the Parkinson’s disease specialist service notified if there was evidence of deterioration or disease progression?
3. Was a swallow screen completed at admission for this patient with Parkinson’s disease?
4. If swallowing difficulties or communication concerns were identified, was the patient referred to Speech and Language Therapy (SLT)?
5. Were dopaminergic medications prescribed and administered on time throughout the admission?
6. Was a dopaminergic medication review completed and documented at discharge?
7. Was the patient’s nutritional status assessed (e.g., MUST score completed)?
8. Were SLT, pharmacy, dietetics, and nutrition teams involved in multidisciplinary team (MDT) discussions when swallowing difficulties were present?
9. Was written information on swallow status and ability to take oral medication included in the discharge summary?

For the purpose of the audit, admission swallow screening was defined in accordance with the NCEPOD recommendation as documented assessment of swallowing at admission, including the patient’s ability to swallow food, fluids and medication, saliva control, and relevant history of pneumonia. This represented an initial clinical screen performed by healthcare professionals assessing the patient at admission and was distinct from a formal Speech and Language Therapy assessment or instrumental swallowing assessment.

Clinical deterioration was defined in accordance with the NCEPOD recommendation as any documented indication of deterioration or progression in the patient’s clinical state identified from the clinical record or discussion with the patient or carers. New or worsening dysphagia was identified where new swallowing difficulty, or deterioration in previously documented swallowing ability, was recorded during the admission.

Timely dopaminergic therapy administration was defined as administration within 30 minutes of the prescribed time, in accordance with the NCEPOD recommendation and NICE Quality Standard 164.

Data analysis

Data were collated and analysed descriptively using Microsoft Excel (Microsoft Corporation, Redmond, WA, USA). Results are presented as frequencies and percentages, with subgroup denominators used where appropriate. Given the descriptive quality-improvement design and small sample size, no inferential statistical testing was performed. Figures were manually created by the authors using Canva (Canva Pty Ltd., Sydney, Australia) and are based on the audit data.

## Results

Over the 20-month audit period, 45 acute admissions of patients with Parkinson’s disease were identified and included in the analysis. Of these, 36% (16/45) were referred from primary care services; however, swallowing status at the point of referral was documented in only 6% (1/16), indicating that information regarding swallowing difficulty was infrequently communicated before hospital admission. Clinical deterioration during admission was identified in 58% (26/45) of patients. Of these, 54% (14/26) were referred to the Parkinson’s disease specialist service, while 46% (12/26) had no documented specialist referral, demonstrating variation in escalation when clinical deterioration was recognised.

Swallow screening at the point of admission was completed in 18% (8/45) of cases (Figure 1A). Among those screened, existing swallowing impairment was identified in 88% (7/8). Documented features included difficulty swallowing food, fluids or medication, impaired saliva control, and recurrent chest infections. During admission, 24% (11/45) developed new or worsening dysphagia; however, only 36% (4/11) were referred to Speech and Language Therapy (SLT), while 64% (7/11) had no documented SLT referral (Figure 1B). These findings are summarised in Figure 1.

**Figure 1:**
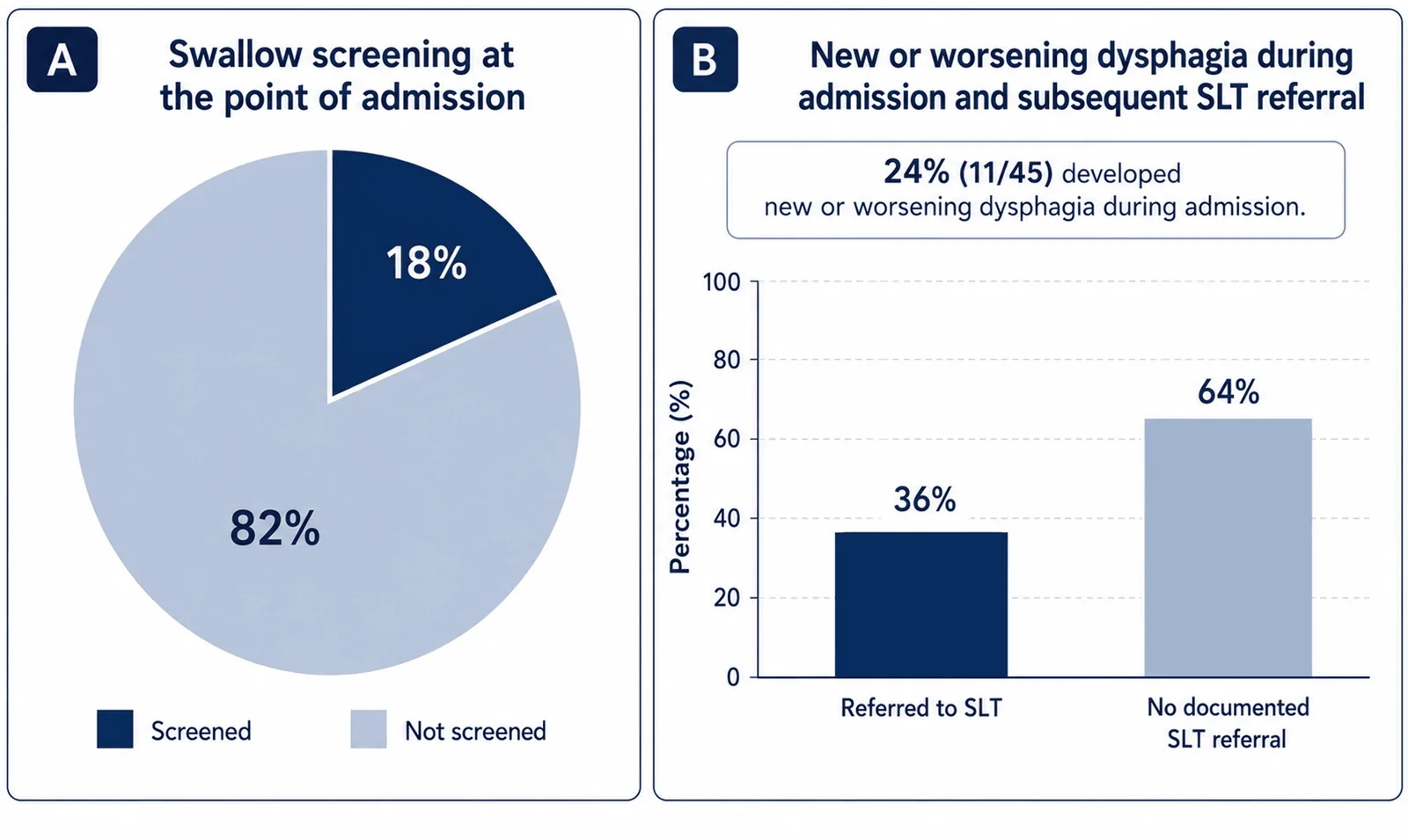
Swallow Screening at Admission and Subsequent SLT Referral (%) (A) Swallow screening at the point of admission was documented in 18% (8/45) of patients, while 82% (37/45) had no documented admission swallow screen. (B) During admission, 24% (11/45) of patients developed new or worsening dysphagia. Among these 11 patients, 36% (4/11) were referred to Speech and Language Therapy (SLT), while 64% (7/11) had no documented SLT referral. Original figure created by authors using audit data.

Timely administration of dopaminergic therapy was well maintained, with 91% (41/45) of patients receiving treatment on schedule throughout admission. Among the 11 patients with swallowing difficulty, alternative medication routes or formulations were documented in 73% (8/11), while 27% (3/11) had no documented adjustment.

Nutritional screening using the Malnutrition Universal Screening Tool (MUST) was completed in 51% (23/45) of admissions. The original audit identified that only one-third of patients considered nutritionally at risk were referred to dietetic services, indicating scope to strengthen the link between identification of nutritional risk and appropriate specialist input.

Multidisciplinary team (MDT) review occurred in 16% (7/45) of cases. All documented MDT reviews included SLT input, with additional involvement from pharmacy in 71% (5/7), dietetics in 43% (3/7), and the nutrition team in 29% (2/7).

Finally, swallowing status or diet-modification advice was documented in only 7% (3/45) of discharge summaries. This indicates that clinically important swallowing recommendations were not consistently communicated at transition from hospital to community care.

Overall compliance with the principal dysphagia-related care standards is summarised in Figure 2.

**Figure 2:**
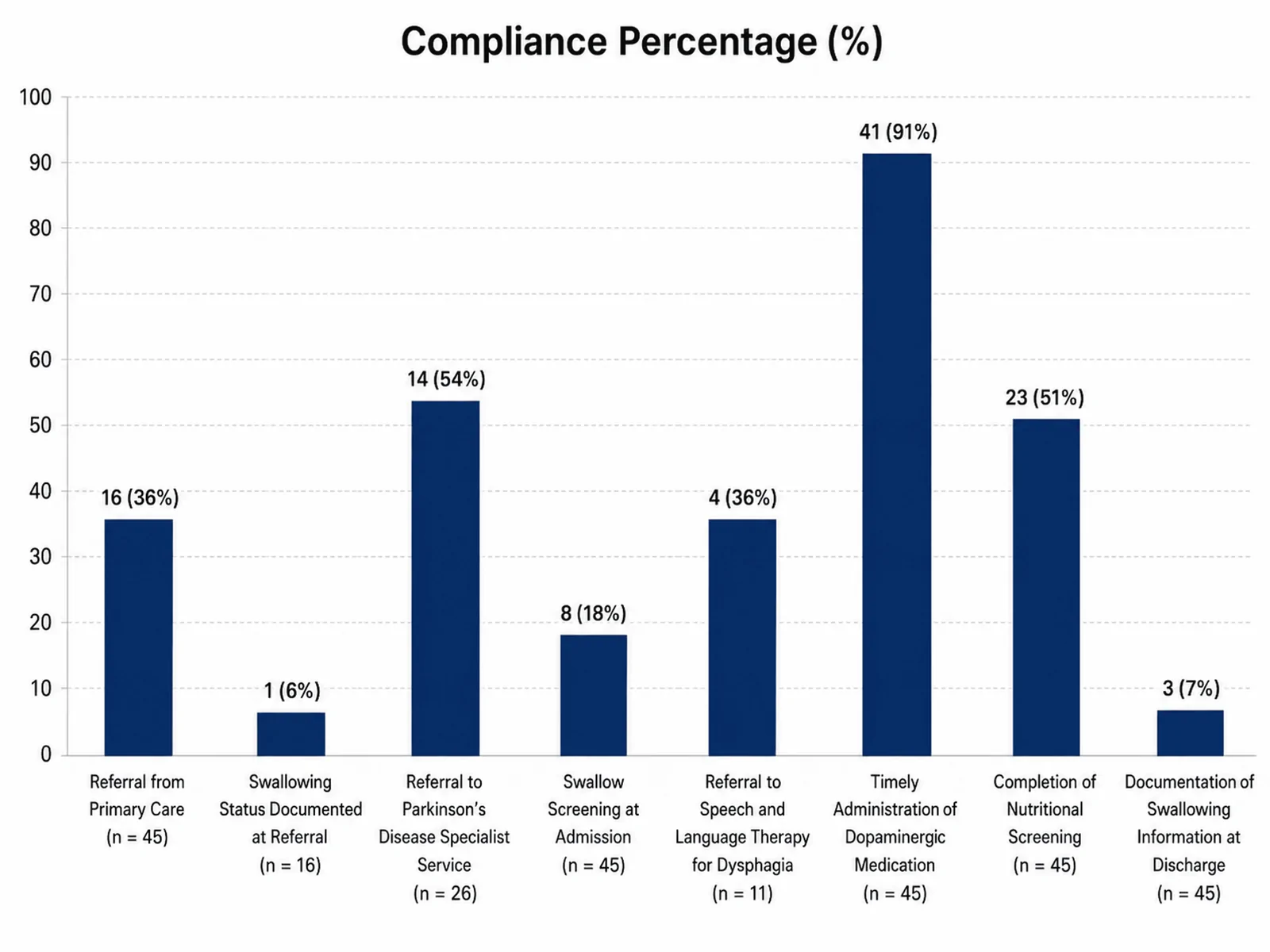
Compliance with Dysphagia-Related Care Standards in Hospitalised Patients with Parkinson’s Disease (%) Bars represent the number and percentage of patients meeting each audited care standard. Percentages are calculated using the relevant denominator for each measure; subgroup denominators apply where appropriate, including swallowing status at referral (n = 16), Parkinson’s disease specialist referral following clinical deterioration (n = 26), and Speech and Language Therapy (SLT) referral among patients with new or worsening dysphagia (n = 11). All other measures are reported against the total audit cohort (n = 45). Original table created by the authors using audit data.

## Discussion

Dysphagia in Parkinson’s disease (PD) is particularly challenging because clinically important swallowing dysfunction may be present even when patients do not describe overt symptoms. The discrepancy between patient-reported symptoms and objectively identified swallowing impairment is well described, and international consensus recommendations therefore advocate active screening rather than relying solely on spontaneous reporting of dysphagia [10]. This vulnerability has a recognised pathological basis: α-synuclein deposition and axonal degeneration have been demonstrated within peripheral motor nerves innervating the pharyngeal musculature in PD [11]. Airway protection may be further compromised by abnormalities in sequential voluntary cough associated with penetration and aspiration risk [12], as well as impaired cough efficacy in this population [13]. Importantly, swallowing function in PD is not static; acute illness and associated clinical deterioration may unmask previously unrecognised dysphagia or exacerbate established swallowing impairment, particularly in the context of infection, reduced mobility, fatigue, altered cognition, reduced oral intake, and disruption of usual dopaminergic therapy [8,10]. This makes reassessment particularly important during an acute hospital admission, even when dysphagia was not previously documented.

These mechanisms provide important context for the findings of our audit. Among the 16 patients referred from primary care, swallowing status was documented in only one referral. Absence of documentation does not establish that dysphagia was present in all remaining patients; however, communication of known swallowing difficulty, recurrent chest infections, problems swallowing medication, or previous Speech and Language Therapy (SLT) recommendations would provide clinically valuable information when an acutely unwell patient enters secondary care. The more striking finding occurred at admission. NCEPOD recommends assessment for dysphagia in all acutely unwell patients with PD, irrespective of the primary reason for admission [8], yet only 18% (8/45) had a documented swallow screen. Seven of these eight patients demonstrated existing swallowing impairment. This finding requires cautious interpretation because the screened group was small and potentially selected and therefore cannot be used to estimate the prevalence of dysphagia across the cohort. Nevertheless, the high proportion of abnormal assessments among those screened illustrates how clinically relevant swallowing impairment may be identified when it is actively sought and reinforces the rationale for incorporating swallowing assessment into routine acute PD care [10].

During admission, 24% (11/45) developed new or worsening dysphagia, but only 36% (4/11) were referred to SLT. Recognition of swallowing difficulty is most useful when it leads to appropriate assessment of swallowing safety and informs decisions regarding food and fluid consistency, compensatory strategies, medication administration, and the need for further investigation [8,10]. Similar challenges have been described in other hospital settings. Martinez-Ramirez et al. reported that swallowing evaluations and aspiration precautions were not routinely implemented among hospitalised patients with PD, with many assessments occurring only after an aspiration event, highlighting the importance of greater clinical awareness and preventive practice [14].

Clinical deterioration was documented in 58% (26/45) of our cohort, while PD specialist input was obtained in 54% (14/26). Acute illness may interact with PD through infection, reduced oral intake, worsening mobility, altered swallowing, and disruption of established medication regimens. Specialist review when deterioration is recognised may therefore assist in identifying these interacting factors and coordinating appropriate management, in keeping with NCEPOD recommendations [8].

In contrast, timely dopaminergic medication administration was an area of strong performance, with 91% (41/45) receiving treatment on schedule. This is particularly important because delayed or omitted dopaminergic therapy can worsen motor function and clinical status [7]. However, among the 11 patients with swallowing difficulty, alternative medication formulations or routes were documented in 73% (8/11). Medication timing and medication delivery are related but distinct aspects of care: a medicine may be prescribed and offered at the correct time while the patient remains unable to swallow that formulation safely or effectively. Medication-related dysphagia is well recognised in PD [6], and identification of swallowing difficulty should therefore prompt consideration not only of when dopaminergic medication is administered but also of how it can be delivered safely.

Nutritional assessment showed further variation, with MUST screening completed in 51% (23/45) of admissions, while 15 patients with documented nutritional concerns had no recorded dietetic referral. Dysphagia may compromise both food and fluid intake and contribute to malnutrition and dehydration [2,10]; nutritional assessment should therefore form part of the same clinical pathway rather than being considered separately from swallowing assessment. Multidisciplinary discussion was documented in 16% (7/45) of admissions. Notably, when MDT review occurred, SLT was involved in all seven cases, with additional pharmacy, dietetic, and nutrition-team input according to clinical need. This reflects the multidimensional nature of dysphagia in PD, where swallowing safety, nutrition, medication administration, and neurological management frequently overlap and benefit from coordinated professional input.

Continuity of this management beyond the hospital admission was less consistently documented, with swallowing recommendations appearing in only 7% (3/45) of discharge summaries. For patients leaving hospital with altered diet or fluid consistency, modified medication formulations, SLT recommendations, or ongoing community follow-up requirements, clear communication is essential to ensure that clinically important decisions made during admission remain visible to primary care, community services, and care providers.

The wider relevance of these process measures is supported by outcome data from hospitalised populations. In a large retrospective cohort of 334,395 non-elective PD admissions, Di Luca et al. found that dysphagia was associated with 44% longer hospital stays and 46% higher inpatient costs, as well as increased odds of aspiration pneumonia and mortality [15]. Similarly, Dilmaghani et al. demonstrated that increasing severity of oropharyngeal dysphagia was associated with shorter time to hospitalisation or death in patients with PD [16]. Collectively, this literature reinforces the clinical importance of recognising dysphagia early and addressing swallowing impairment before complications become established. Although early screening alone cannot be assumed to improve these outcomes, timely recognition creates an opportunity for appropriate assessment and intervention at an earlier stage of the acute admission.

The findings from this first audit cycle have therefore informed a focused programme of quality improvement within our acute medical setting. A ward- and Emergency Department-based educational poster, “A SIP Test Today Keeps Aspiration Away”, has been developed as a visual prompt to increase awareness of swallowing assessment in acutely unwell patients with PD and to reinforce appropriate documentation and escalation (Figure 3). A structured four-question dysphagia screening tool (4QT) is also planned for incorporation into the assessment proforma for acutely unwell patients with PD, providing a brief and practical prompt to identify possible swallowing difficulties requiring further assessment [17].

**Figure 3:**
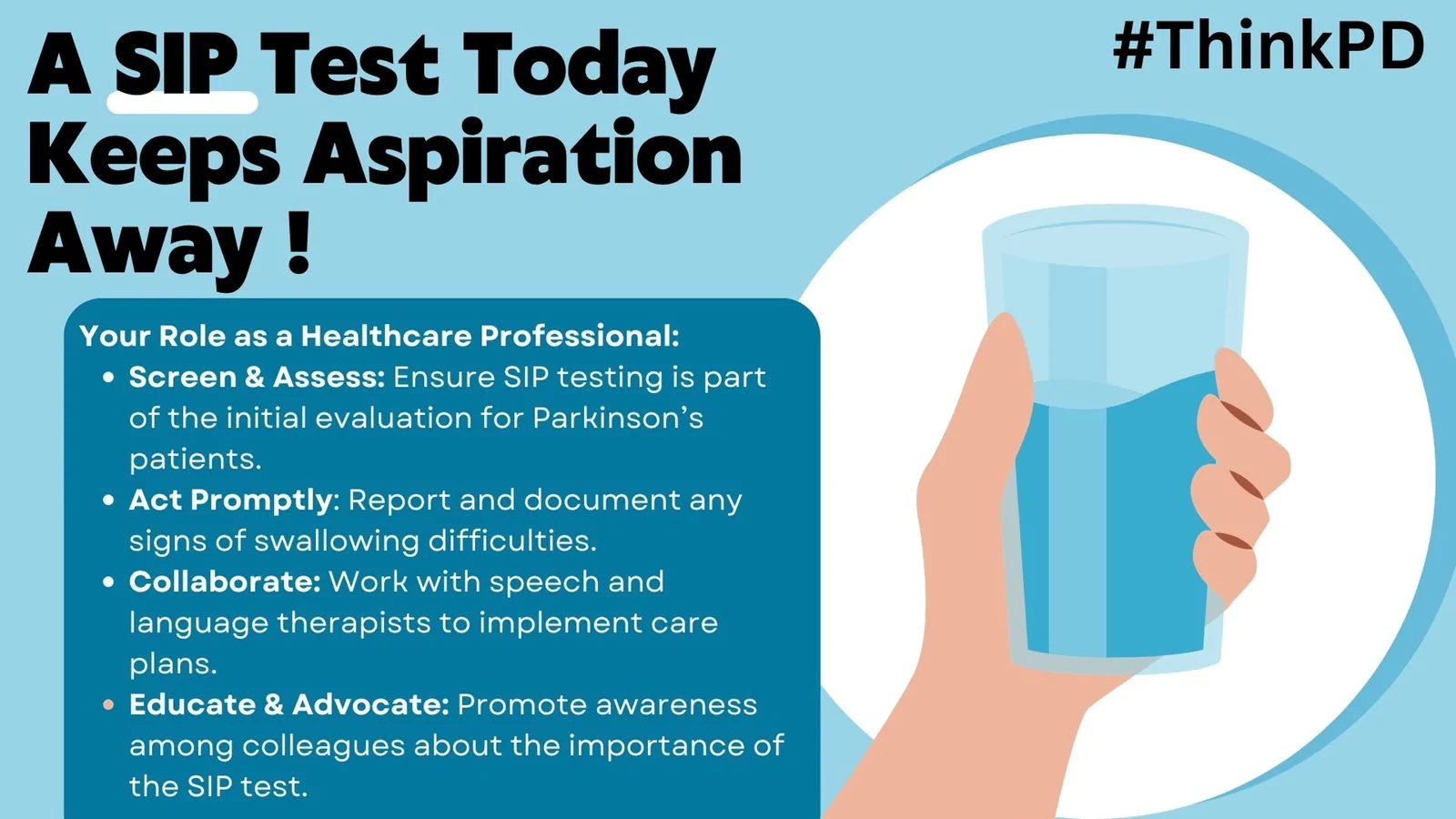
Ward-based educational poster developed as part of the quality-improvement intervention to increase awareness and prompt swallowing assessment in acutely unwell patients with Parkinson’s disease. Image credit: Original figure manually created by Aparna Ravikumar using Canva (Canva Pty Ltd., Sydney, Australia).

These measures will be supported by targeted education for medical, nursing, and allied healthcare professionals addressing less overt manifestations of dysphagia, indications for SLT and PD specialist involvement, safe administration of dopaminergic medication, nutritional assessment, and communication of swallowing recommendations. The intention is not simply to increase completion of a screening measure, but to establish a reliable clinical pathway in which recognition of swallowing impairment leads to timely assessment and appropriate multidisciplinary action. Following implementation, a subsequent audit cycle will reassess the same standards to determine whether improvements are achieved in screening, specialist referral, medication and nutritional review, multidisciplinary involvement, and discharge communication.

Ultimately, earlier recognition and coordinated management of dysphagia aim to preserve safe nutrition and medication delivery, reduce potentially preventable aspiration-related morbidity, and support better outcomes for acutely unwell patients with Parkinson’s disease.

Limitations

The findings of this audit should be interpreted within the context of a retrospective quality-improvement evaluation conducted in a single district general hospital and may therefore not be directly generalisable to hospitals with different patient populations or clinical practices. This first audit cycle was primarily designed to assess processes of care and establish a baseline for improvement rather than to evaluate causal associations with outcomes such as aspiration pneumonia, length of stay, readmission, or mortality. Despite these limitations, the audit provides a valuable assessment of real-world clinical practice, identifies specific areas for service improvement, and establishes a baseline against which future change can be measured. Re-audit following implementation of the planned interventions will provide an opportunity to assess improvement over time and, where feasible, incorporate relevant clinical outcome measures.

## Conclusions

Dysphagia may be clinically subtle in Parkinson’s disease, yet its consequences extend across airway protection, nutrition, medication administration, functional recovery, and continuity of care. This audit identified variation at several interconnected stages of the acute-care pathway, including admission screening, SLT and PD specialist involvement, adaptation of medication formulations, nutritional assessment, multidisciplinary management, and communication at discharge. At the same time, the high compliance rate of timely dopaminergic medication administration demonstrates that reliable adherence to important PD-specific standards is achievable within routine clinical practice.

The findings provide clear and measurable priorities for the next quality-improvement cycle. Introducing structured dysphagia screening, visible clinical prompts, and multidisciplinary staff education aims to make consideration of swallowing a consistent part of the assessment of acutely unwell patients with PD and, importantly, to ensure that recognised concerns lead to timely assessment and appropriate intervention. By strengthening the pathway from recognition through multidisciplinary management to discharge communication, the wider aim is to deliver safer, more coordinated care and reduce preventable complications associated with unrecognised or inadequately managed dysphagia. Re-audit following implementation will determine whether these interventions result in measurable and sustainable improvement across the dysphagia care pathway.
